# Gene expression profile of *Aedes aegypti* females in courtship and mating

**DOI:** 10.1038/s41598-019-52268-5

**Published:** 2019-10-29

**Authors:** Diego Peres Alonso, Melina Campos, Heitor Troca, Rafael Kunii, Frédéric Tripet, Paulo Eduardo Martins Ribolla

**Affiliations:** 1 Sao Paulo State University – UNESP, Biotechnology Institute and Bioscience Institute, Botucatu, 18618-689 Brazil; 2 Keele University, Centre for Applied Entomology and Parasitology, Keele, ST5 5BG UK

**Keywords:** Behavioural ecology, Gene expression, Transcriptomics

## Abstract

*Aedes aegypti* is the most synanthropic and anthropophilic mosquito of Culicidae. This species always cohabits with humans and is extremely opportunistic. Vector dispersal is directly related to the ability of the females on successfully finding a mate in a generally patchy urban scenario. In the present work, we investigate transcriptional changes in *Ae. aegypti* females during the courtship process and after mating. We observe a substantial alteration in gene expression triggered just upon contact with *Ae. aegypti* males, which in turn was not fully correlated to the changes triggered by the contact. After analysing shared significant differentially regulated genes between conspecific contact and insemination, the major part of the observed transcriptomic change triggered by contact is reversed after mating, indicating an intermediary situation between naive and mating conditions that we hypothesize to be crucial for mating success. Upon contact, several chemosensory related genes are repressed, especially odorant binding proteins. Most of these genes return to higher expression rates after mating. None of these genes are significantly regulated by the encounter of a different species, *Aedes albopictus*. The results presented here might be applied to an innovative control approach focusing on the semiochemical systems of mosquitoes in an effort to disrupt undesirable host–insect interaction to reduce the risk of pathogen transmission to humans.

## Introduction

*Aedes* (Stegomyia) *aegypti* Linnaeus is a mosquito of major medical importance due to its broad global distribution and its role as the main vector of various arboviral diseases, such as dengue, chikungunya, urban yellow fever and Zika viruses. It is a cosmopolitan species, found mainly in the tropics, with more than 60% of the human population at risk of arboviruses transmitted by this species^1,2^.

*Ae. aegypti* is the most synanthropic and anthropophilic species of the Culicidae family. It always cohabits with humans and as a result is considered a particularly opportunistic species. A lack of urban infrastructure that ultimately favours the production and maintenance of breeding sites, a high degree of adaptation to different environmental conditions and eggs that are highly resistant to desiccation contribute to the successful spread and establishment of this mosquito. The vector is known to be extremely well adapted to urban environments with most of its breeding sites found close to human dwellings. *Ae. aegypti* females also prefer man-made, artificial containers for laying eggs, even very small ones, such as plastic cups, PET bottles, aluminum cans and small plant pots^3^. Females also exhibit, under both field and laboratory conditions, the so-called “skip oviposition” behaviour, which leads them to lay eggs across several breeding sites^4,5^. This particular behaviour is thought to contribute to mosquito dispersion when suitable breeding sites are not available for the female, forcing them to search for new oviposition sites based on the availability of food, sun exposure or water^6^. In a recent study on *Ae. aegypti* oviposition behaviour, it was demonstrated that a single female may distribute eggs among up to 11 different breeding sites^7^. This is a major characteristic that shapes the overall pattern of the potential breeding site distribution, leading to highly fragmented “breeding units”, as even a small number of eggs originating from a few adults may be enough to permit local persistence of mosquito populations. An immediate consequence of this pattern of fragmented breeding site distribution is that successful mating may be constrained by females successfully encountering a mate.

Although wing beats are considered the major acoustic signal for attracting males for mating, other cues might be important during this process^8^. It is well known that olfactory cues have a determinant role on human host detection, as well as on nectar sources and oviposition site localization. In this context, aggregation pheromones, sex pheromones, cuticular hydrocarbons and other chemical cues have a major role in courtship and mating behaviour^9^.

In this study, we aim to evaluate the impact of courtship, triggered by the close proximity contact of conspecific and heterospecific (*Aedes albopictus*) males, on the overall *Ae. aegypti* female gene expression. We also assess the gene expression profile related to conspecific insemination in order to transcriptionally compare insemination and courtship processes on *Ae. aegypti* females. We believe that gaining insight into the processes driving mate choice or avoidance during courtship and mating, as well as revealing their underlying mechanisms and chemical cues, might allow the design of alternative control methods for these mosquito vectors.

## Material and Methods

### Mosquito rearing

*Ae. aegypti* and *Ae. albopictus* laboratory strains were recently established from eggs collected in Botucatu city, Sao Paulo State, Brazil (22°53′S 48°26′W) in 2015, using ovitraps. The rearing was conducted in a climate-controlled insectarium maintained at 28 °C and 70% relative humidity, with a 12 h light:12 h dark photoperiod. Firstly, eggs from the field were transferred to a tray (27 cm × 19 cm × 7 cm) containing 1 L of water to hatch. After hatching, larvae were fed with ground fish food until the pupae stage. Species and gender were determined in adults by examination of the scutum and antennae. Adults were placed in screened cages (30 cm × 30 cm × 30 cm) and fed with 10% sterile sucrose solution, which was replaced every 3 days.

### Experimental groups

For the experiments, males and females of both species were kept in different cages from the pupae stage. When the larvae changed to the pupae stage, we started to separate each pupa between males and females. The sexual identification was made by analyzing the last segment of the abdomen from the ventral part of the individual, with males having the last segment elongated and wide, while females have the same last segment thinner and short^10^. Twenty pupae from each sex and species were put separately into small trays of 8 × 5 × 3 cm containing water and then transferred to a screened cage of 30 × 30 × 30 cm. Pilot experiments were then performed to determine the mean time length for 100% insemination of *Ae. aegypti* and *Ae. albopictus* females in each conspecific crossing, putting together two males with twenty females. In these conditions, the mean time length for 100% insemination was 90 min for *Ae. albopictus* and 20 min for *Ae. aegypti*. These results helped us to define a safe time window for the females entering in contact with males and not being inseminated, therefore we used these times as standards also for heterospecific crosses.

**Figure 1:**
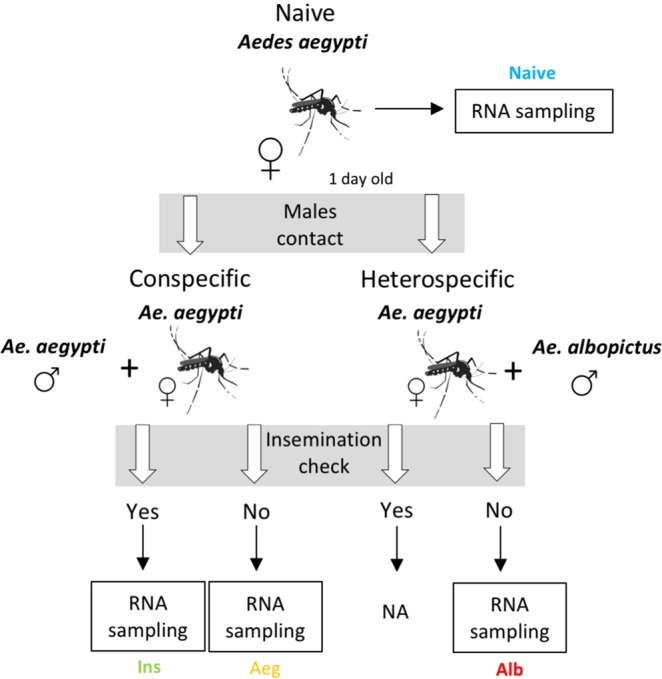
Experimental design. Workflow showing stage and condition when RNA from heads and thoraxes of *Ae. aegypti* females were sampled.

*Ae. aegypti* females were submitted to four experimental conditions: (i) no contact with males (Naive); (ii) contact with *Ae. aegypti* males (Aeg); (iii) contact with *Ae. albopictus* males (Alb); (iv) inseminated by *Ae. aegypti* males (Ins) (Fig. 1). We used three cages for each one of the four experimental conditions as biological replicates. All mosquitos in the experiments were used 24 h after emergence from pupae. In addition, all females were anesthetised in ice and dissected, separating the head and thorax from abdomen. The insemination status was checked by dissecting female spermathecae on clean microscope slides, according to Gary and collaborators^11^.

The four groups were obtained as follows: (a) Naive: each experimental cage of this group had five naive females that had never had contact with males of any species. We checked the spermatheca of all females and they were negative for the presence of spermatozoa. Three pools each containing the heads and thoraxes of five individuals were put into 1.5 mL microtubes containing 500 µL of TRIzol® and then stored at −80 °C in a freezer. This pool preparation approach was carried out for every experimental group described below. (b) Aeg: In each experimental cage, we put together two *Ae. aegypti* males and twenty *Ae. aegypti* females that had never had contact with adult males. We removed five females from the cage during four consecutive intervals of 5 min. This continued until we obtained a total of five females (one pool) that were found not inseminated after spermatheca checking. Therefore, a maximum of 20 min of contact was allowed. When sperm was found in the spermatheca, these females were discarded and not used to compose the pool of this group. (c) Alb: In each experimental cage, we put together two Ae. albopictus males and twenty Ae. aegypti females that had never had contact with adult males. We removed five females from the cage during six consecutive intervals of 15 min. As we could not find any inseminated females by checking the spermatheca after 90 min of contact, we used five females from the first interval for pooling. (d) Ins: In each experimental cage, we put together two *Ae. aegypti* males and twenty *Ae. aegypti* females that had never had contact with adult males. We removed all females after 30 min in the cage and checked the spermatheca to form a pool of five inseminated females.

### RNA-Seq library preparation and sequencing

Total RNA was extracted from head and thorax pools of *Ae. aegypti* females using the TRIzol® reagent protocol according to the manufacturer’s instructions (ThermoFisher Scientific, Waltham, USA). RNA concentrations were quantified in each sample using a Qubit^TM^ 2.0 fluorometer (ThermoFisher Scientific, Waltham, USA). The levels of RNA degradation were assessed by a 1% agarose gel. cDNA libraries were constructed with 200 ng of total RNA using the SureSelect Strand Specific RNA Library Prep Kit (Agilent Technologies, Santa Clara, USA) following the manufacturer’s instructions. Library products were then sequenced using an Illumina Nextseq platform (Illumina, San Diego, USA) on a single-end 150 bp run.

### RNA-Seq data processing and differential gene expression analysis

A CLC Genomics Workbench 7.01 platform was used to remove the adapter and assess the reads quality from the raw reads. The same platform was used to subject reads to mapping to the *Ae. aegypti* predicted transcriptome AaegL5.2 available at VectorBase (https://www.vectorbase.org/organisms/aedes-aegypti). Only unique gene reads count were used for posterior analysis (Supplementary Table 1). Differential expression analysis was performed with DESeq2 package version 1.24.0^12^. Genes presenting an adjusted *p*-value lower than 0.05 were considered as significantly regulated. Principal component analysis (PCA) data visualisation and plotting were performed in R using the pcaExplorer package^13^. Clustering analysis was performed using Euclidean distances and complete linkage method.

### Gene ontology and pathway annotation

Gene ontology analysis was carried out for the identified differential expression gene lists using g:Profiler (https://biit.cs.ut.ee/gprofiler/gost). A Fisher exact test was considered as the statistical method (p-value < 0.05) and the Benjamini & Hochberg method^14^ was used for false discovery rate correction (<0.05).

### Chemosensory related gene family identification

The gene expression profiles of five chemosensory related proteins families were individually analysed. Identification of gene ID and classification was performed using Interpro IDs, i.e., for chemosensory proteins (CSPs, IPR005055), ionotropic receptors (IRs, IPR001320), odorant binding proteins (OBPs, IPR006170), odorant receptor (ORs, IPR004117) and sensory neuron membrane proteins (SNMPs, IPR002159).

## Results

### General sequencing analysis

To understand the transcriptional changes of *Ae. aegypti* females due to courtship and insemination by conspecific (*Ae. aegypti*) and heterospecific (*Ae. albopictus*) males, we performed RNA sequencing (Illumina) libraries for each condition. Here, *Ae. aegypti* females without contact with any males, and therefore not inseminated, were considered as the Naive group. There was no evidence of insemination by *Ae. albopictus* males, consequently three conditions were considered besides Naive females: conspecific courtship (Aeg), heterospecific courtship (Alb) and conspecific insemination (Ins). Three biological replicates per condition were sequenced, resulting in ~19.3 million reads on average per library of which ~10.3 million were mapped to the assembly of the *Ae*. *aegypti* transcriptome (AaegL5). A total of 16,721 genes were identified in the libraries prepared after removal of low expressed genes (global counts <12). Raw reads are deposited at the NCBI BioProject database under the accession number PRJNA551490.

### Unsupervised data clustering and general expression profile

**Figure 2:**
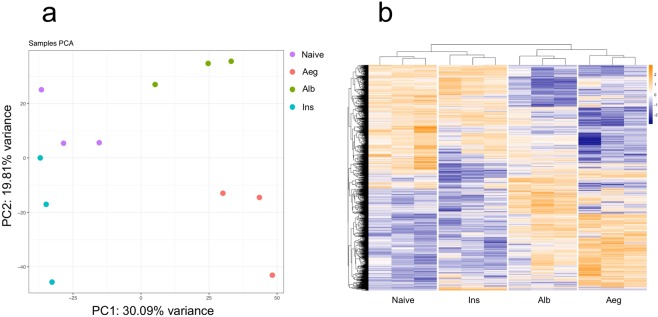
Principal component analysis (PCA) and heatmap of log expression values. Clustering methods using full log expression data set of the three replicates for four *Ae. aegypti* female conditions: Naive (naive), conspecific male’s contact (Aeg), hetero-specific male’s contact (Alb) and inseminated (Ins). (**a**) PCA of the three biological replicates of each condition. (**b**) Heatmap showing expression profile for 1,725 genes that were found to be significantly regulated in at least one of the comparisons using naive females as baseline condition. Expression values were normalized by genes. Clustering analysis was performed using Euclidean distances and complete linkage method.

The full filtered dataset was subjected to PCA and hierarchical clustering (Fig. 2a,b). Both showed consistent results for the three biological replicates. Overall, the first axis (PC1), which accounts for 30% of the observed gene expression variation in *Ae. aegypti* females, separated the libraries into two groups: with male contact (Aeg and Alb) and Naive/inseminated libraries. The second PCA axis (PC2–20%) then separated the heterospecific male contact (Alb) and Naive libraries from the two other conditions. Gene expression profile after male contact is depicted through a comparative heatmap of four treatment groups (Fig. 2b). Together, these results suggest that Naive *Ae. aegypti* females, upon contact with males, alter the expression of a determined set of genes. This expression status, after insemination, changes back to a pattern very similar to the basal Naive condition.

### Differential expression genes in Ae. aegypti females after male contact

**Figure 3:**
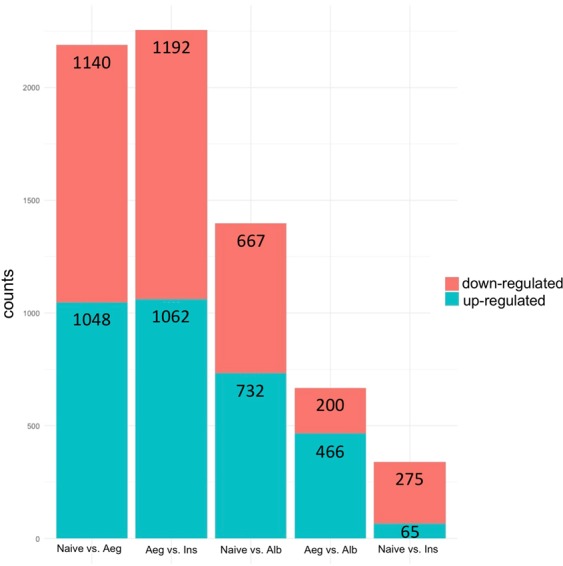
Number of significant differentially expressed genes for all analyzed comparisons. The numbers inside the bars represent total counts of differentially regulated genes per comparison. Blue bar represents up-regulated genes, red bar represents down-regulated genes.

**Figure 4:**
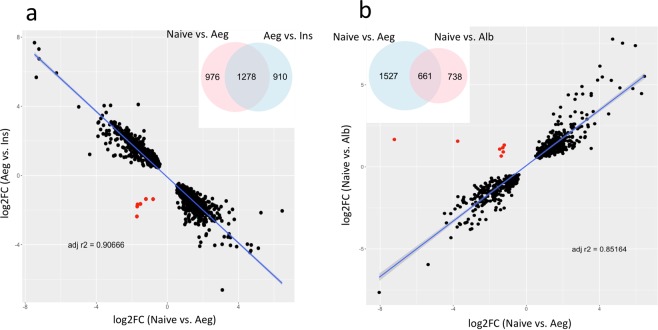
Correlation profile of gene log2FC between conspecific contact and insemination (**a**) and conspecific and heterospecific contact (**b**). Scatter plot comparison of log2FC values of shared DE genes (intersection of Venn diagrams) in both (Naive vs. Aeg) × (Aeg vs. Ins) (**a**) and (Naive vs. Aeg) × (Naïve vs. Alb) (**b**). The insets show Venn diagrams of shared significantly regulated genes. Red dots indicate genes that do not follow the overall correlation pattern observed (negative correlation in 4a; positive correlation in 4b).

The identification of differential expression genes between conspecific/hetero-specific male contact and Naive groups was performed. Significance was defined using an adjusted *p*-value of <0.05. *Ae. aegypti* females in contact with conspecific males (Aeg vs. Naive) presented greater gene regulation compared to *Ae. albopictus* male contact (Alb vs. Naive). A total of 1,048 genes were shown to be up-regulated in *Ae. aegypti* male contact, whereas there were only 732 genes in *Ae. Albopictus*. Similarly, 1,140 genes were found to be down-regulated for the conspecific male contact while heterospecific accounted for 667 genes. Fewer genes were significantly differentially expressed for Aeg vs. Alb, a total of 466 genes were up-regulated and 200 were down-regulated (Fig. 3). Interestingly, when conspecific contact is compared to heterospecific contact (Alb vs. Aeg), we can observe a positive correlation between significant differentially expressed genes with 661 shared significant regulated genes. A total of 1,527 genes was found exclusively in Aeg and 738 in Alb (Fig. 4b).

### Differential expression genes in Ae. aegypti females after insemination

In general, the gene expression profile of inseminated *Ae. aegypti* females was similar to the Naive group (Ins vs. Naive). However, the inseminated group showed a higher number of regulated genes than Naive when using *Ae. aegypti* male contact as the baseline (Fig. 3). Surprisingly, direct comparison between the Naive and inseminated libraries showed very few regulated genes, with 65 up-regulated and 275 down-regulated. When conspecific contact is compared to inseminated females (Aeg vs. Ins), we found a strong negative correlation with 1,278 shared significant regulated genes. A total of 976 genes was found exclusively in AEG and 910 in INS (Fig. 4a).

### Functional analysis of significantly regulated genes

Significantly regulated genes from Aeg vs. Naive, Alb vs. Naive and Aeg vs. Ins comparisons were subjected to analysis using the g:Profiler web server^15^ for functional analysis. Genes were ordered by log2FC and significance was defined using *p*-values of <0.05 after Bonferroni correction for the three classes of gene ontology (GO), which are molecular function, the biological process and the cellular component. The GO analysis of regulated genes for Aeg vs. Naive revealed two significant terms: *Odorant Binding* and *Arginine Metabolic Process*. For Alb vs. Naive, ten GO terms were found enriched: *Structural Constituent of Cuticle, Arginine Metabolic Process, Alpha-amino acid Metabolic Process, Respirasome* and six others related to cellular respiration. For Aeg vs. Ins, 25 GO terms were found enriched: *Respirasome, Catalytic activity, acting on a protein*, another 14 related to general cellular respiration and another nine related to endo and exopeptidase activity evidencing general proteolysis (Table 1).

**Table 1:** Gene ontology (GO) enrichment analysis for significantly regulated genes in conspecific contact (Naïve × Aeg), heterospecific contact (Naïve × Alb) and insemination (Aeg × Ins).

		Term name	Term ID	Adjusted P value
Naïve × Aeg	MF	odorant biding	GO:0005549	0.00869554
	BP	arginine metabolic process	GO:0006525	0.00285098
Naïve × Alb	MF	NADH dehydrogenase (ubiquinone) activity	GO:0008137	0.00153504
	MF	oxidoreductase activity, acting on NAD(P)H, quinone or similar compound as acceptor	GO:0016655	0.00153504
	MF	NADH dehydrogenase (quinone) activity	GO:0050136	0.00153504
	MF	NADH dehydrogenase activity	GO:0003954	0.00250215
	MF	structural constituent of cuticle	GO:0042302	0.00467783
	MF	oxidoreductase activity, acting on NAD(P)H	GO:0016651	0.01965961
	BP	arginine metabolic process	GO:0006525	0.00799965
	BP	alpha-amino acid metabolic process	GO:1901605	0.0155279
	BP	ATP metabolic process	GO:0046034	0.01614061
	CC	respirasome	GO:0070469	0.04291496
Aeg × Ins	MF	proton transmembrane transporter activity	GO:0015078	6.9377E-06
	MF	peptidase activity, acting on L-amino acid peptides	GO:0070011	1.4114E-05
	MF	peptidase activity	GO:0008233	1.6708E-05
	MF	endopeptidase activity	GO:0004175	0.00016708
	MF	serine-type endopeptidase activity	GO:0004252	0.0007364
	MF	serine-type peptidase activity	GO:0008236	0.00123631
	MF	serine hydrolase activity	GO:0017171	0.00123631
	MF	ATPase activity, coupled to transmembrane movement of ions, rotational mechanism	GO:0044769	0.00231902
	MF	catalytic activity, acting on a protein	GO:0140096	0.00292244
	MF	monooxygenase activity	GO:0004497	0.00430074
	MF	exopeptidase activity	GO:0008238	0.00618993
	MF	carboxypeptidase activity	GO:0004180	0.03070018
	MF	oxidoreductase activity, acting on paired donors, with incorporation or reduction of molecular oxygen	GO:0016705	0.03694957
	BP	ATP metabolic process	GO:0046034	2.9711E-06
	BP	proton transmembrane transport	GO:1902600	0.00057526
	BP	respiratory electron transport chain	GO:0022904	0.00454189
	BP	proteolysis	GO:0006508	0.00470694
	BP	cellular respiration	GO:0045333	0.01237147
	BP	oxidative phosphorylation	GO:0006119	0.01588846
	BP	ATP biosynthetic process	GO:0006754	0.03503251
	BP	energy coupled proton transport, down electrochemical gradient	GO:0015985	0.03503251
	BP	ATP synthesis coupled proton transport	GO:0015986	0.03503251
	CC	respirasome	GO:0070469	5.30E-07
	CC	proton-transporting two-sector ATPase complex	GO:0016469	0.00203989
	CC	respiratory chain complex	GO:0098803	0.00327362

MF: Molecular Function, BP: Biological Process and CC: Cellular Content.

### Expression profile of chemosensory gene families

**Figure 5:**
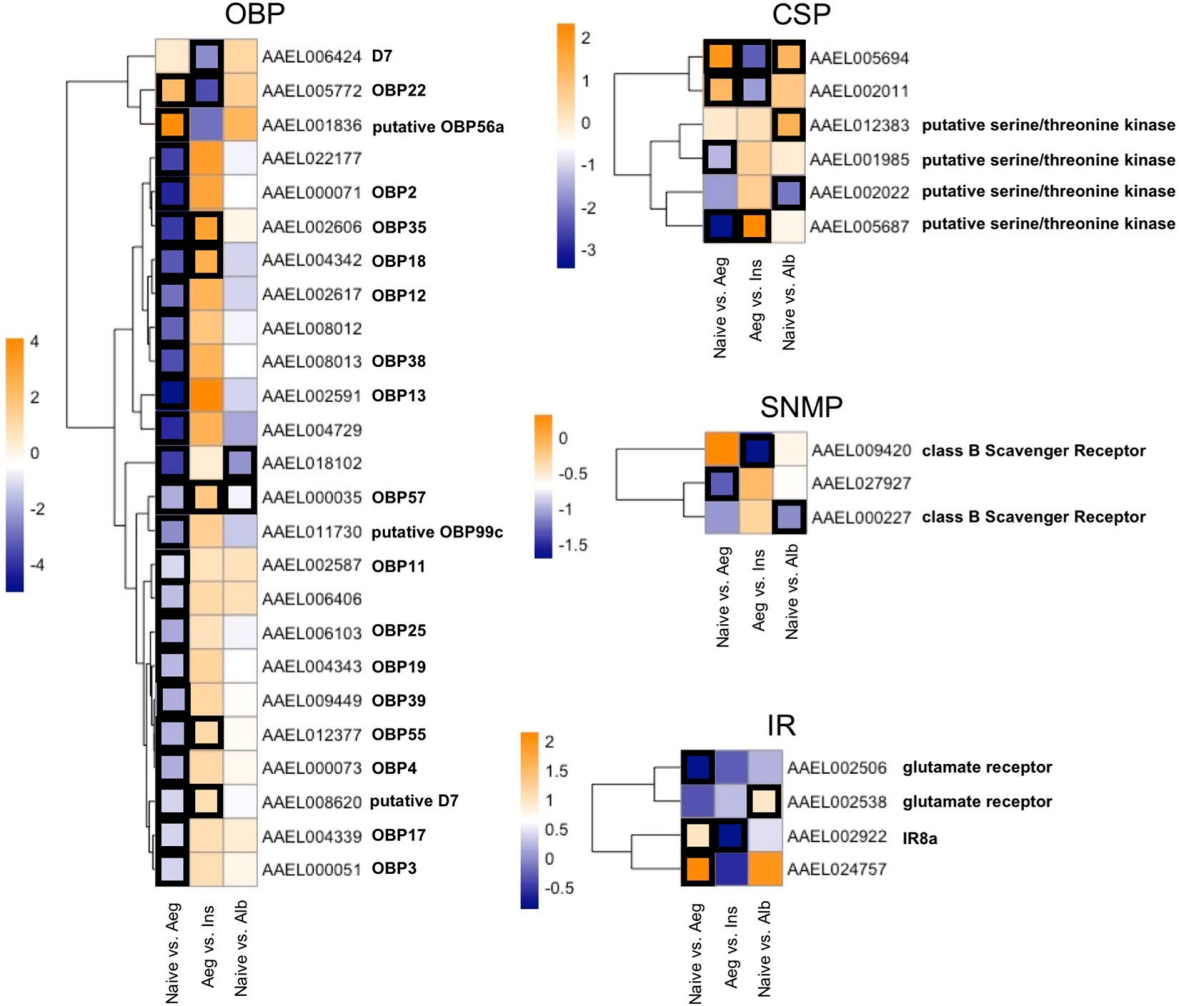
Heatmap of log2FC of chemosensory related gene families. Black boxes represent significantly regulated genes in each comparison. Only genes that were found significantly regulated for at least one of the comparisons are shown. OBP – odorant binding protein; CSP – chemosensory protein; SNMP - sensory neuron membrane protein; IR – ionotropic receptor.

Assuming that chemosensation is an essential sensory factor in mosquitoes to locate mates and hosts, we investigated the expression profiles of five chemosensory-related proteins families, namely, CSPs, IRs, OBPs, ORs and SNMPs (Fig. 5). A total of 65 OBPs were identified out of the 16,721 expressed genes here characterized. Apart from two genes (*OBP22* and *putative OBP56a*), all genes suggested significant down-regulation between conspecific male contact and Naive females. Among them, 24 genes showed significant log2FC after multiple test correction. However, the *Ae. albopictus* male’s courtship did not alter this set of genes in the same way. For the SNMPs, nine genes were identified; one was found significantly down-regulated in conspecific male contact with Naive (AAEL027927); one in the inseminated females (*SCRBQ1*) and one in hetereospecific contact (*SCRB8*). We identified 38 genes in the CSP family, among them, two were significantly up-regulated with conspecific contact and down-regulated after insemination; (AAEL005694 and AAEL002011). AAEL005694 is also significantly up-regulated upon heterospecific contact. Two other genes (AAEL005687 and AAEL001985), which are putative serine/threonine kinase, presented significant down-regulation for Aeg vs. Naive comparison. AAEL005687 is found to be significantly up-regulated in the inseminated females. Two other putative serine/threonine kinase genes (AAEL12383 and AAEL002022) were significantly regulated only after heterospecific contact. Twenty-two IRs were identified, among them, three (*Ir8a*, *glur5(AAEL002922)* and AAEL02757) were significantly regulated by conspecific contact but not by heterospecific contact, with *Ir8a* also being regulated by insemination. Another *glur5(AAEL002538)* is regulated only by heterospecific contact. We could identify 63 ORs but none of them were significantly regulated in any comparison (Fig. 5).

## Discussion

In the present work, we investigated transcriptional changes in *Ae. aegypti* females during the courtship process with both *Ae. aegypti* and *Ae. albopictus* males and after conspecific mating. Following our experimental approach for heterospecific crosses, we could not observe heterospecific mating (satyrisation) after 90 min of contact. It is well documented that in both laboratory and field conditions, *Ae. albopictus* males are capable of inseminating *Ae. aegypti* females; however, this process only seems to occur after long periods of heterospecific contact (~3 weeks) in laboratory conditions. Moreover, the geographic origin of *Ae. albopictus* males might determine the satyrisation potential, which might be the case for our field population^16^.

We observed a substantial alteration in the gene expression triggered just upon contact with *Ae. aegypti* males, which in turn was distinct from changes triggered by contact with *Ae. albopictus* males (Fig. 2a,b). Interestingly, a significant number of regulated genes triggered by conspecific contact are reversed after mating in a switch-like manner: when a gene is found up-regulated in one condition, in the other condition it is present down-regulated and vice-versa. From 1,278 shared significant regulated genes between conspecific contact (Aeg) and insemination (Ins), only six do not follow this pattern (Fig. 4a). Conversely, when we compare conspecific contact against heterospecific contact (Alb vs. Aeg), we observe only 661 shared significant regulated genes, with 1,527 genes found exclusively in Aeg and 738 in Alb (Fig. 4b). It is noteworthy that the expression pattern shows a positive correlation for these two datasets; with the exception of 7 out 661, shared genes are regulated in a similar fashion: when a gene is found up-regulated in one condition, in the other condition it is also found up-regulated, with the same being true for down-regulated genes. These results suggest an overlap of the gene expression profile between these two conditions, reflecting core genes that are regulated upon contact regardless of male species.

To further investigate the overall differential gene expression profile, we performed a GO analysis for the heterospecific courtship (Naive vs. Alb), insemination (Aeg vs. Ins) and conspecific courtship (Naive vs. Aeg) processes (Table 1). GO analysis of significantly regulated genes in the heterospecific courtship dataset revealed ten enriched terms, with seven of them related to general cellular respiration, two related to the bioprocessing of amino acids and one term named structural constituent of cuticle. Interestingly, cuticular hydrocarbons play a central role in the chemical communication of insects. As they are usually non-volatile, they are mainly detected by contact^9^. Although this term is related to the structural features of the cuticle, they might have a role in the efficient detection of specific cuticular hydrocarbons by the heterospecific male. In the GO analysis of the insemination process, we could find 25 enriched terms, 15 of them are related to general cellular respiration and ten of them are related to endo and exopeptidase activity evidencing general proteolysis. This result is in agreement with a previous study assessing mating-induced transcriptome changes in the reproductive tract of female *Ae. aegypti*^17^. Even though we did not analyze reproductive tissues in our study, the proteolysis profile was found to be very similar. In the GO analysis of the conspecific courtship, we could find two enriched terms: *Odorant Binding and Arginine Metabolic process*. The Odorant Binding term is comprised of genes coding for proteins related to olfaction that are usually involved in foraging, courtship, mating and the choice of oviposition. As a major representative group of these molecules, OBPs have been described as important components in the recognition of odours^18^. The Arginine Metabolic process term is comprised of genes related to the general bioprocessing of arginine with a major role on the urea cycle and was also found in the heterospecific courtship.

Taken together, these findings point towards an intermediary state between naive and mating conditions. To our knowledge, this is the first time that such an intermediary condition has been disclosed and we can hypothesize that it is crucial for mating success. Once we found the Odorant Binding term enriched throughout this intermediary state, which in turn reflects conspecific contact, we decided to investigate the expression profiles of five chemosensory related proteins families for the above-mentioned processes (Fig. 5).

From the overall analysis, we can draw the assumption that upon contact, several genes that may be related to host seeking are repressed, especially OBPs. Most of these genes return to higher expression rates after mating. Out of twenty-five differentially expressed OBP genes only two were found to be significantly regulated by the encounter of a different species, namely, *Ae. albopictus*. Interestingly, the inseminated females presented an overall gene expression profile very similar to the naive ones, which is also true for the OBP related genes. Conversely, we find an IR co-receptor (IR8a) significantly up-regulated by conspecific contact and down-regulated by insemination but not significantly regulated by heterospecific contact. These types of co-receptors form an odor-responsive ion channel with other odor-tuned IRs and are reported to detect volatile chemicals in the environment. IR8a appears to be strongly associated to host seeking behavior since it was recently shown that this co-receptor is required for mosquito attraction to lactic acid, a major component of human sweat^19^. In this case the up-regulation upon conspecific contact seems to contribute to an early priming of females for a future host seeking, reflecting a more complex interplay between genes related to courtship and mating in *Ae. aegypti*.

In general, insects can use a myriad of chemical cues, such as animal odours and pheromones, to encounter each other and also to detect plant nectar or vertebrate hosts^20^. These compounds are generally small hydrophobic molecules, which enter the sensory organs passing across the sensillum lymph surrounding the olfactory neurons. This passage is thought to be facilitated by OBPs, which are capable of transporting these compounds to the insect ORs^21,22^. Interestingly, Cabrera and Jaffe^23^ showed that in laboratory conditions, swarming *Ae. aegypti* males, settled upwind in an olfactometer evoked female flight activity. More importantly, it was observed that test females reacted with flight to the odours produced by male mosquitoes, but not to the presence of odours from a rat. When the females detected the odours from a rat, they flew upwind and clustered at the area of the olfactometer where the air stream hit the cage and attempted to probe to find a blood meal. If both odours were present at the same time, the females presented the characteristic flight, suggesting that the male’s odour elicited behaviour that overrode that produced by the host. This clearly indicates that when females can sense the presence of a male, they shift from their host seeking behaviour to mating behaviour, regardless of the perception of host cues. Taking the present results into account, we can draw the hypothesis that conspecific contact with males, or at least male perception by *Ae. aegypti* females, is an important cue for triggering a mating response and preparing females for insemination. From our point of view, this may be achieved by down-regulation of OBPs capable of switching off female host seeking behaviour.

By extrapolating these results to field conditions, we put forward a scenario to illustrate the importance of courtship in the mating behaviour of *Ae. aegypti* females. Once *Ae. aegypti* possess the so-called skip oviposition behaviour, this can shape the distribution pattern of potential mosquito breeding sites, leading to highly fragmented “breeding units”. As a direct consequence of this pattern, encounters between males and females tend to be rarer, thus compromising the mating process. Thus, females need to be able to respond to male stimuli in a mandatory fashion, ignoring any other cues that are not related to male perception.

Mosquito chemical ecology is generally accepted as a major theme of investigation on which upcoming vector-borne disease control strategies may rely on. The results presented here might be applied to an innovative control approach based on mosquito chemical communications where we could develop molecular strategies (i.e. target mutagenesis in OBP related genes) to lock females in “mating mode”, rendering them less effective in finding a host for blood meal and consequently reducing the transmission of vector-borne diseases.

Finally, we hope that this dataset will serve as an important resource to guide the selection of candidate genes involved in mosquito courtship and mating behaviour to be precisely targeted mutated with now available CRISPR-Cas9 tools, allowing the development of stable engineered mosquito lines to test the function of candidate genes.

## Supplementary information

Supplementary Dataset 1
